# *LIM Homeobox 4* (*lhx4*) regulates retinal neural differentiation and visual function in zebrafish

**DOI:** 10.1038/s41598-021-81211-w

**Published:** 2021-01-21

**Authors:** Rui Guo, Kangkang Ge, Yuying Wang, Minxia Lu, Fei Li, Lili Tian, Lin Gan, Donglai Sheng

**Affiliations:** 1 College of Life Sciences, Zhejiang University, Hangzhou, 310013 Zhejiang China; 2 Key Laboratory of Organ Development and Regeneration of Zhejiang Province, College of Life and Environmental Sciences, Hangzhou Normal University, Hangzhou, 311100 Zhejiang China; 3 Hangzhou Jingbai Biotechnology Co, LTD, Hangzhou, 310004 Zhejiang China; 4 Traditional Chinese Medicine Pharmacy, Zhejiang Hospital, Hangzhou, 310007 Zhejiang China

**Keywords:** Developmental neurogenesis, Development

## Abstract

LIM homeobox 4 (LHX4) is expressed in the photoreceptors (PRs) of the outer nuclear layer (ONL) and bipolar cells (BCs) of the inner nuclear layer (INL) in mouse and chicken retina. It regulates the subtype-specific development of rod BCs and cone BCs in the mouse retina. However, no report has been published on its expression and function in the zebrafish retina. In this study, we assessed the expression of Lhx4 using in situ hybridization (ISH) technique and explored its role in zebrafish (*Danio rerio*) retinal development via morpholino (MO) technology. We found that the expression of *lhx4* in the zebrafish retina begins 48 h post-fertilization (hpf) and is continuously expressed in the ONL and INL. A zebrafish model constructed with *lhx4* knockdown in the eyes through vivo-MO revealed that: *lhx4* knockdown inhibits the differentiation of Parvalbumin^+^ amacrine cells (ACs) and Rhodopsin^+^ rod photoreceptors (RPs), enhances the expression of visual system homeobox 2 (*vsx2*); and damages the responses of zebrafish to light stimulus, without affecting the differentiation of OFF-BCs and rod BCs, and apoptosis in the retina. These findings reveal that *lhx4* regulates neural differentiation in the retina and visual function during zebrafish embryonic development.

## Introduction

As an ideal model, the vertebrate retina is highly conserved and has been employed to explore the regulatory mechanisms of related genes during the development of the central nervous system (CNS)^1–3^. In the mature retina, six types of neurons and one type of glial cell are orderly distributed, forming a five-layered structure, which includes three distinct nuclear layers and two different plexiform layers^4, 5^. Photoreceptors (PRs), including cone photoreceptors (CPs) and rod photoreceptors (RPs), are located in the outer nuclear layer (ONL); horizontal cells (HCs), bipolar cells (BCs), amacrine cells (ACs), and Müller glial cells (MGCs) are positioned in the inner nuclear layer (INL); whereas the retinal ganglion cells (RGCs) are situated in the ganglion cell layer (GCL)^6^. The functions of these retinal cells co-ordinated. Dysplasia of retinal cells can impair visual conduction.

During retinal development in zebrafish (*Danio rerio*), pluripotent retinal progenitor cells (RPCs) gradually exit the cell cycle and generate different cell types in a spatiotemporal pattern^7^. Retinal neurogenesis involves a series of biological events regulated by various genes, such as paired box 6 (*pax6*)^8^, visual system homeobox 2 (*vsx2*)^9, 10^, LIM homeobox 2 (*lhx2*)^11^, atonal homolog 5 (*ath5*)^12, 13^, and cone-rod homeobox (*crx*)^14, 15^, and extracellular signal pathways, including Sonic hedgehog^16^, Wnt^17, 18^, Notch^19, 20^, and FGF^21 signaling^ pathways.

Most of the *lhx* genes exert critical functions in regulating retinal development. For instance, lhx1/lhx5 contributes to retinal neurogenesis during early development^22, 23^. As a marker of RGCs, LIM homeobox 1 (Isl1) is essential for the differentiation and survival of RGCs, co-ordinated by brain-specific homeobox/POU domain protein 3B (Brn3b)^24, 25^. Besides, Isl1 and Lhx1 jointly regulate the migration and morphogenesis of HCs subtypes during the later retinal development^26^. Lhx2 is responsible for the specialization of the eye area, the morphogenesis of the optic cup, and the development of ACs and MGCs in the mature retina^27–29^. Of note, Lhx9 is vital for the development of nitric oxide-synthesizing (NOs) ACs subtype^30^. Lhx3 and Isl2 are associated with enhanced maturation of PRs in the retina, particularly when their expression is impeded^31^. LHX4 is essential for the development of the pituitary, spinal motoneurons, and retina^32–34^. During retinal development, Lhx4 is expressed in the PRs and BCs of INL in the retina of mouse and chicken^35–38^. It is crucial in regulating the subtype-specific development of rod BCs and cone BCs in the mouse retina^34^. However, no reports have been published on the expression and function of Lhx4 during zebrafish retinal development.

Here, we provide a first-time report on *lhx4* expression during zebrafish retinal development by in situ hybridization (ISH). Furthermore, we performed a morpholino (MO) knockdown experiment in zebrafish and examined the effects of *lhx4* knockdown in zebrafish eyes during retinal neural differentiation and light stimulus responses.

## Results

### *lhx4* expression in the ONL and INL during retinal development of zebrafish

**Figure 1:**
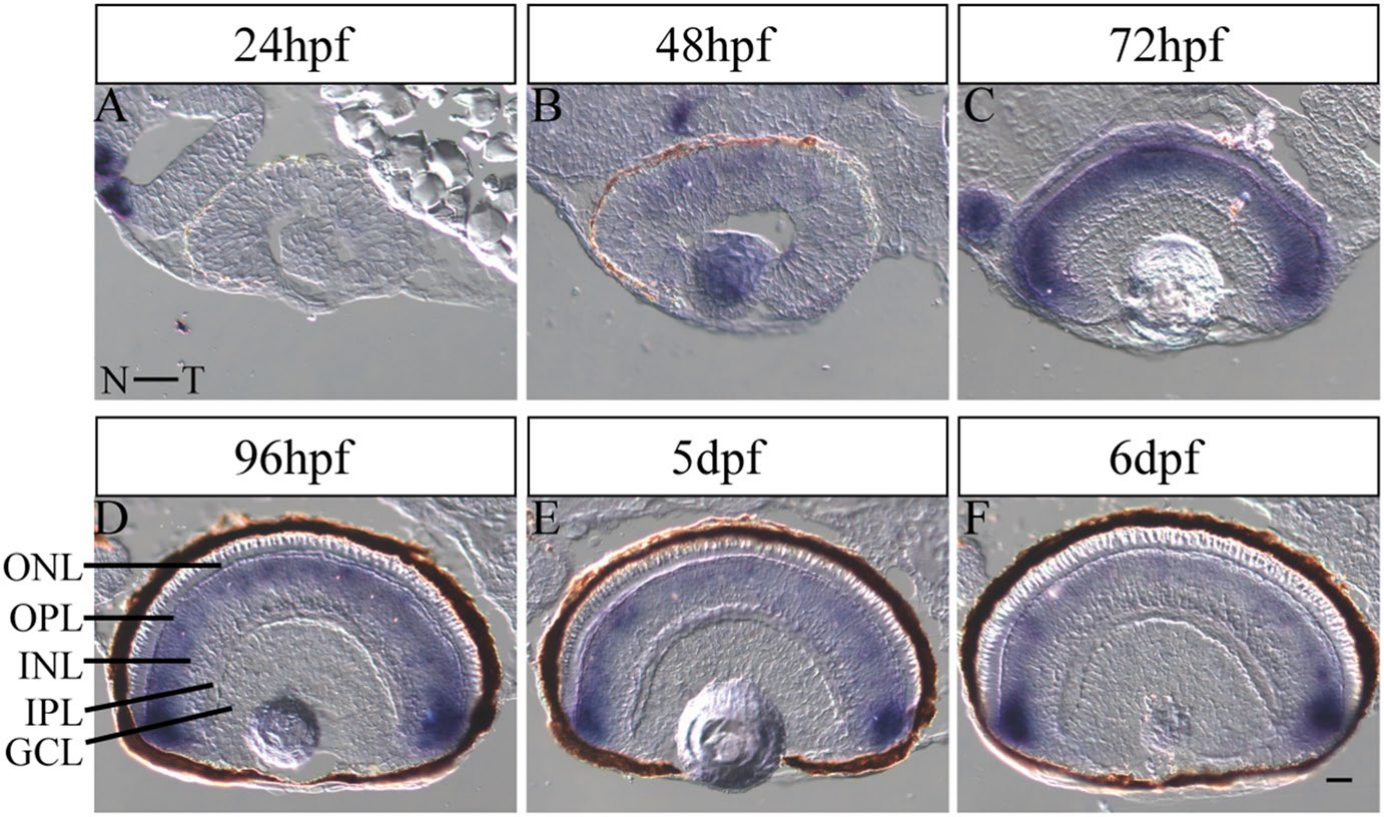
Expression pattern of *lhx4* in zebrafish retinal development. All figures are horizontal sections along the temporal-nasal axis (T-N). (**A**) The expression of *Lhx4* was not detected in the retina at 24 hpf. (**B**) The expression of *Lhx4* in the dorsal-nasal of the retina was weak at 48 hpf. (**C**) *Lhx4* was expressed in the ONL and INL at 72 hpf. (**D**–**F**) The expression of *Lhx4* in the ONL and INL was gradually weakened, and the signal in the CMZ was relatively strong from 96 hpf to 6 dpf. *ONL,* outer nuclear layer; *INL,* inner nuclear layer; *GCL,* ganglion cell layer; *OPL,* outer plexiform layer; *IPL,* inner plexiform layer. Scale bar = 20 μm.

To assess the temporal and spatial expression of *lhx4* during retinal development in zebrafish, ISH was conducted from 24 hpf to 6 dpf. Notably, ISH results showed that: a weak *lhx4* signal in the nasal retina at 48 hpf (Fig. 1A,B). At 72 hpf, when retinal differentiation was complete, the *lhx4* signal was enhanced, but the location was limited in the ONL and INL (Fig. 1C). From 96 hpf to 6 dpf, the signal was gradually weakened in the ONL and INL, whereas the signal was relatively strong in the ciliary marginal zone (CMZ) (Fig. 1D–F). The findings demonstrated that lhx4 might play a role in zebrafish retinal development.

### *lhx4* knockdown in the eyes of zebrafish

In zebrafish, *lhx4* is located on chromosome 8, with one transcript (2627 bp of total length) and six exons. It encodes a transcription factor (Lhx4) containing 391 amino acids. Splice-blocking *lhx4* MO (4MO) and *lhx4 *vivo-MO (4vMO) were conducted to evaluate the function of *lhx4* in zebrafish retinal development. Notably, the designed MOs blocked exon 2 (E2) splicing. The primers used to detect the MOs knockdown efficiency are located in E1 and E4 of the lhx4 transcript (Fig. S1A).

When 4MO or control MO (CTMO) were injected into the yolk sac at the 1- to 4-cell stages, the dose–response experiment showed that the optimal injection dose of 4MO was 2 ng. At this concentration, embryos showed no severe deformity, with a clear mutation band (band 2, 379 bp) detected by RT-PCR, while *lhx4* mRNA in WT and CT embryos only produced band 1 (551 bp) (Fig. S1B). Sequencing results showed that the sequences of band 1 from the embryos of all groups were similar. However, band 2 produced by 4MO embryos was 172 bp shorter than band 1 (Fig. S1D). The 172-bp sequence was the E2 of *lhx4* mRNA. These findings suggest that 4MO can effectively exclude the E2 of *lhx4* transcript. However, through *lhx4* knockdown by 4MO, we found that the phenotypes of zebrafish embryos were similar to human combined pituitary hormone deficiency (CPHD), characterized by a deficiency of the growth hormone and retardation (Figs. S2–S7). Therefore, we concluded that this model is unsuitable for exploring the function of lhx4 during retinal development.

To solve this drawback, we injected 4vMO or control vivo-MO (vCTMO) in the left eyes at 26–27 hpf at a maximum dose (5 ng). The mutation band 2 of *lhx4* mRNA in both 4vMO left and right eyes could be detected via RT-PCR (Fig. S1C,D). This demonstrated that 4vMO injected in the left eyes could exclude E2 of *lhx4* mRNA in both the left and right eyes. We further examined the effects of 4vMO on the morphological development of zebrafish. Based on the results, 4vMO had no significant impact on zebrafish morphology and eye development (Fig. S8). Thus, we validated the successful construction of the zebrafish model with *lhx4* knockdown in the eyes via 4vMO.

### Effects of *lhx4* knockdown on retinal neural differentiation

Here, we adopted immunofluorescence staining and qRT-PCR to detect the expression of different cell markers in the zebrafish retina.

**Figure 2:**
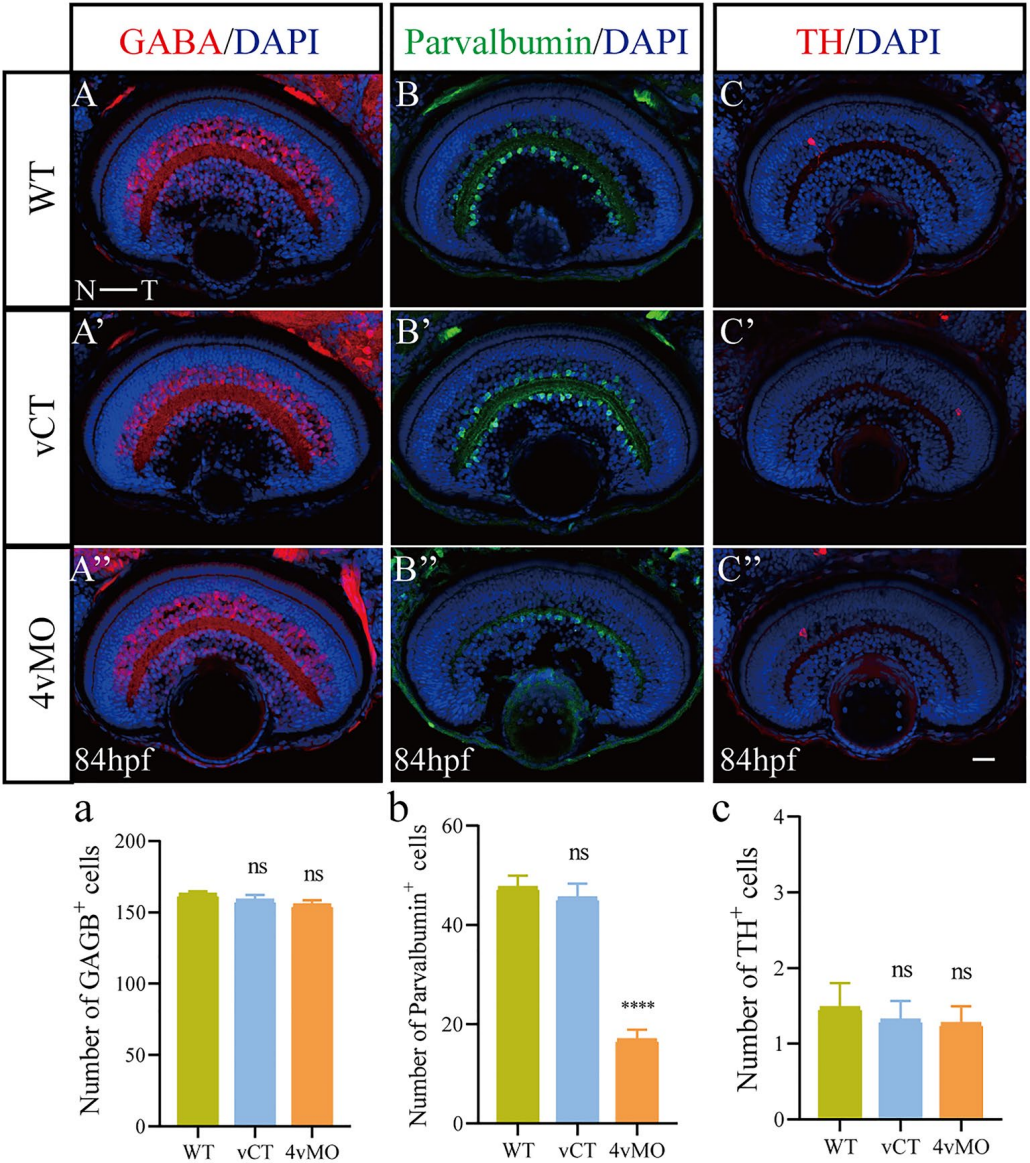
Effects of *lhx4* knockdown via vivo*-*MO in the eyes on the ACs differentiation. All figures are horizontal sections along the temporal-nasal axis (T-N). (**A**–**C’’**) Immunofluorescence staining with different ACs markers, GABA, Parvalbumin, and Calretinin in WT, vCT, and 4vMO retinas at 84 hpf. Blue, DAPI staining of the nuclei. Scale bar = 20 μm. (**a**–**c**) Statistical analysis of GABA^+^, Parvalbumin^+^, and Calretinin^+^ cells in WT, vCT, and 4vMO retinas at 84 hpf. ns, *P* > 0.05; ****, *P* < 0.0001; vCT vs. WT; 4vMO vs. vCT. Results are presented as the mean ± SEM (n ≥ 10).

At 84 hpf, many GABA^+^ ACs, but a small number of Parvalbumin^+^ ACs and TH^+^ ACs were observed in WT (Fig. 2A–C) and vCT (Fig. 2A’–C’) retinas. Compared to WT and vCT groups, the expressions of GABA and TH in the retina of 4vMO group showed no significant change (Fig. 2A’’,C’’), though Parvalbumin expression decreased significantly (Fig. 2B’’). Compared to WT and vCT groups, the number of GABA^+^ and TH^+^ AC in the retina of 4vMO group showed no significant difference (*P* > 0.05, n ≥ 10, Fig. 2a,c), whereas the number of Parvalbumin^+^ ACs decreased significantly (*P* < 0.0001, n ≥ 10, Fig. 2b).

**Figure 3:**
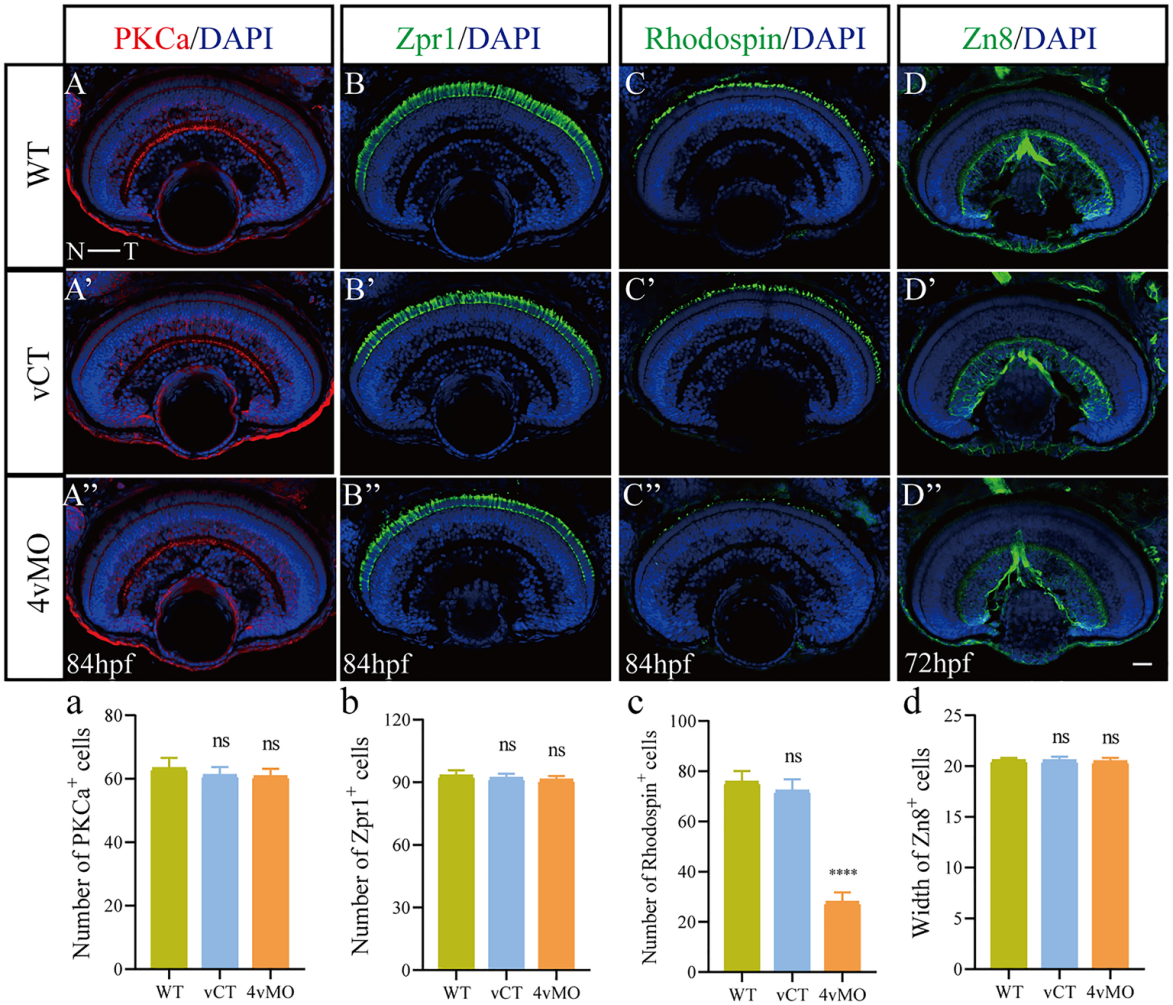
Effects of *lhx4* knockdown via vivo*-*MO in the eyes on the other retinal neuronal differentiation. All figures are horizontal sections along the temporal-nasal axis (T-N). (**A**–**D”**) Immunofluorescence staining with other retinal neural markers, PKCα (at 84 hpf), Zpr1(at 84 hpf), Rhodopsin (at 84 hpf), and Zn8 (at 72 hpf) in WT, vCT, and 4vMO retinas. Blue, DAPI staining of the nuclei. Scale bar = 20 μm. (**a**–**d**) Statistical analysis of PKCα^+^, Zpr1^+^, Rhodopsin^+^, and Zn8^+^ cells in WT, vCT, and 4vMO retinas. ns, *P* > 0.05; ****, *P* < 0.0001; vCT vs. WT; 4vMO vs. vCT. Results are presented as the mean ± SEM (n ≥ 10).

At 84 hpf, Zpr1 and Rhodopsin were expressed in the ONL (except for CMZ) in WT (Fig. 3B,C) and vCT (Fig. 3B’,C’) retinas. Notably, Zpr1 expression in 4vMO retina was not significantly changed (Fig. 3B’’), though Rhodopsin expression decreased significantly (Fig. 3C’’). The number of Zpr1^+^ CPs in 4vMO retina was not significantly different from that in WT and vCT retinas (*P* > 0.05, n ≥ 10, Fig. 3b), whereas the number of Rhodopsin^+^ RPs was reduced considerably (*P* < 0.0001, n ≥ 10, Fig. 3c). However, at 84 hpf, the INLs in WT, vCT, and 4vMO retinas (Fig. 3A-A’’) were characterized by many PKCα^+^ BCs. A continuous axon layer of PKCα^+^ BCs was visible in the inner plexiform layer (IPL). Furthermore, there was no significant difference in the number of PKCα^+^ cells among the groups (*P* > 0.05, n ≥ 10, Fig. 3a). At 72 hpf, the GCLs in WT (Fig. 3D) and vCT (Fig. 3D’) retinas were developed, and there were Zn8^+^ RGCs in the whole GCL. Compared to WT and vCT groups, the expression of Zn8 in the retina of 4vMO group showed no significant change (Fig. 3D’’). We also reported no significant difference in the width of GCL among the three groups (*P* > 0.05, n ≥ 10, Fig. 3d).

**Figure 4:**
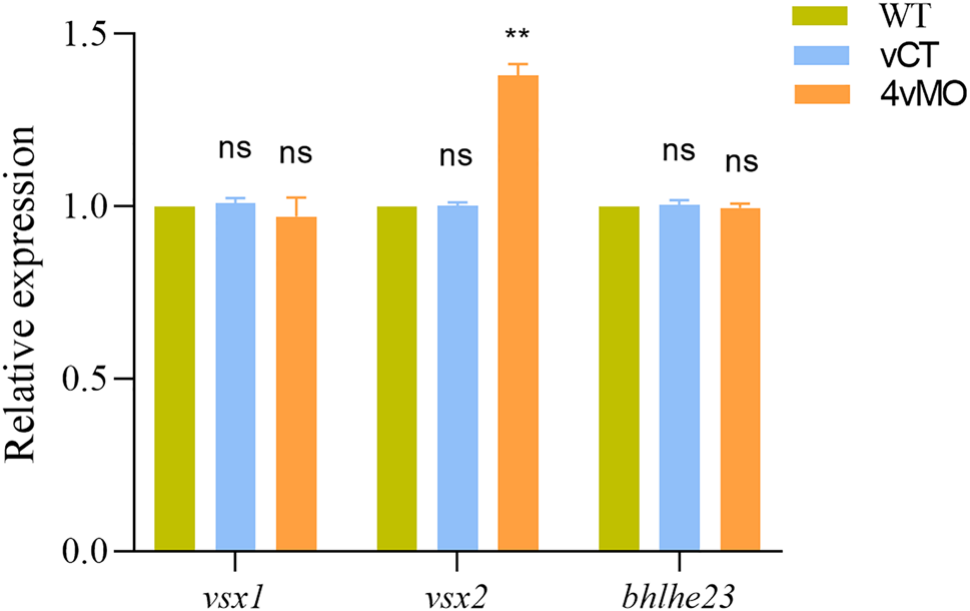
Effects of *lhx4* knockdown via vivo*-*MO in the eyes on the expression of *vsx1*, *vsx2*, and *bhlhe23* at 60 hpf. ns, *P* > 0.05; **, *P* < 0.01; ***, *P* < 0.001; ****, *P* < 0.0001; vCT vs. WT; 4vMO vs. vCT. Results are presented as mean ± SEM (n = 3 biological replicates).

Based on the qRT-PCR results, compared to WT and vCT groups, the expression of *vsx1* and *bhlhe23* in the eyes of 4vMO group showed no significant change at 60 hpf (*P* > 0.05), whereas the expression of *vsx2* increased significantly (*P* < 0.01, n ≥ 3, Fig. 4).

Although *lhx4* knockdown via 4vMO did not affect the development of GABA^+^ ACs, TH^+^ ACs, PKCα^+^ BCs, Zpr1^+^ CPs, and Zn8^+^ RGCs, it inhibited the development of Parvalbumin^+^ ACs in the INL and Rhodopsin^+^ RPs in the ONL and enhanced *vsx2* expression in the eyes.

### Effects of *lhx4* knockdown on retinal cell apoptosis

**Figure 5:**
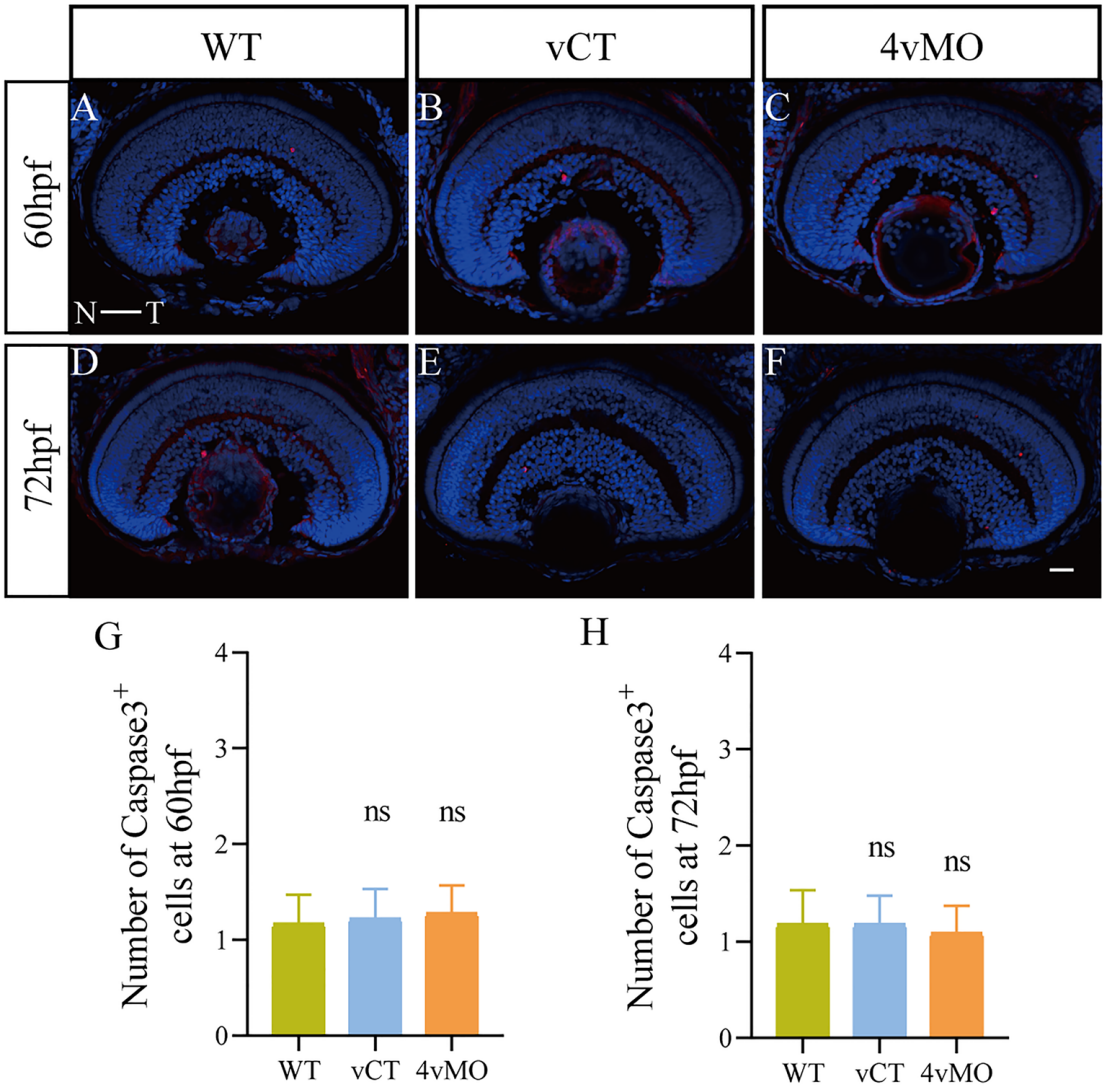
Effects of *lhx4* knockdown via vivo*-*MO in the eyes on the apoptosis in the retina. All figures are horizontal sections along the temporal-nasal axis (T-N). (**A**–**F**) Immunofluorescence staining with Caspase3 at 60 hpf and 72 hpf. Blue, DAPI staining of the nuclei. Scale bar = 20 μm. (**G**,**H**) Statistical analysis of the number of Caspase3^+^ cells in WT, vCT, and 4vMO retinas at 60 hpf and 72 hpf. ns, *P* > 0.05; vCT vs. WT; 4vMO vs. vCT. Results are presented as the mean ± SEM (n ≥ 10).

To determine whether apoptosis potentially decreased Parvalbumin^+^ ACs and Rhodopsin^+^ RPs in the retina, we detected apoptotic cells in the retina using activated Caspase-3 antibody. Immunofluorescence staining results revealed a few apoptotic cells labeled with Caspase-3 in WT (Fig. 5A,D) or vCT (Fig. 5B,E) retinas at 60 hpf and 72 hpf. Notably, the expression of Caspase-3 in 4vMO retina (Fig. 5C,F) was not significantly different from WT and vCT retinas. There was no significant difference in the number of apoptotic cells among the groups (*P* > 0.05, n ≥ 10, Fig. 5G,H). However, massive apoptosis was observed in the retina of larvae treated with 2 mM H_2_O_2_ for 6 h (Fig. S9). These findings demonstrated that *lhx4* knockdown through 4vMO exerts no apoptotic effect in the zebrafish retina.

### Effects of *lhx4* knockdown on the response to light stimulus

**Figure 6:**
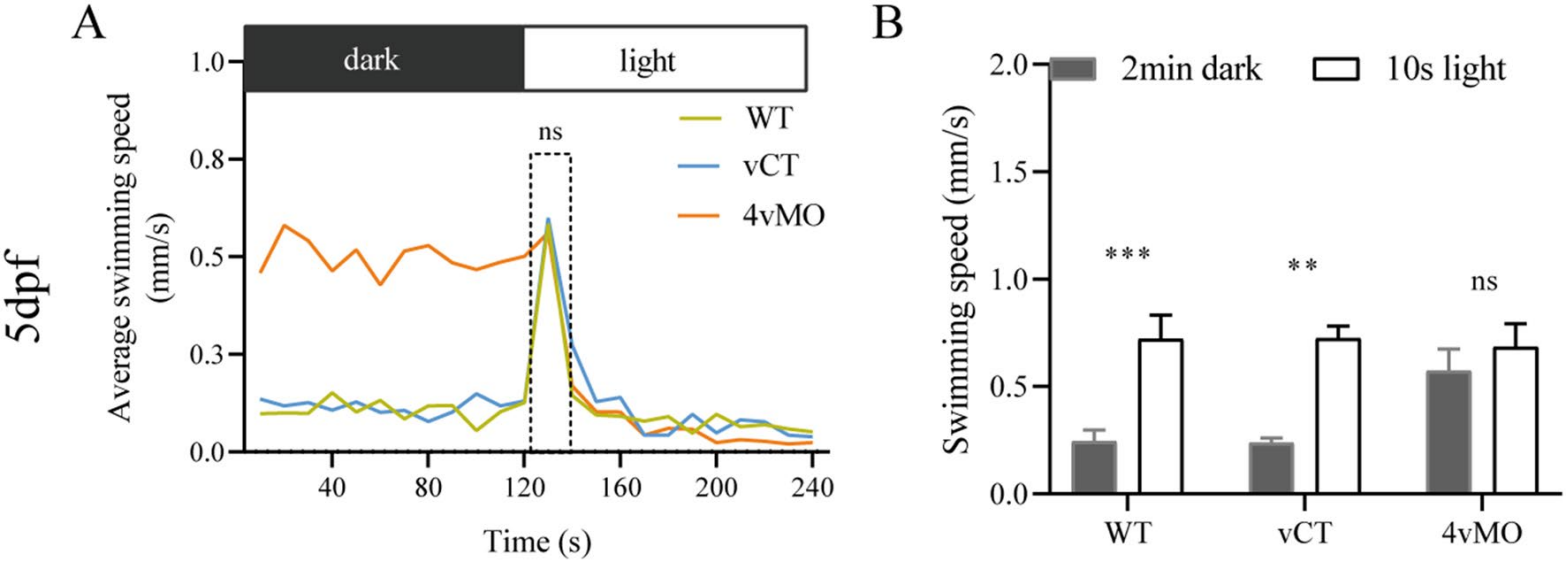
The response of zebrafish larvae to the light stimulus at 5 dpf. (**A**) The average swimming speed of the larvae during the last 2 min dark and 2 min light period. (**B**) The swimming speed during the last 2 min dark and the first 10 s of the 2 min light period. ns, *P* > 0.05; **, *P* < 0.01; ***, *P* < 0.001; vCT vs. WT; 4vMO vs. vCT. Results are presented as the mean ± SEM (n ≥ 40).

We tested the responses of three groups to light stimulus at 5 dpf to explore the effect of *lhx4* knockdown on the visual function of zebrafish using 4vMO. The light stimulation was performed after 2 min of dark treatment (before 30 min of dark pretreatment). The average swimming speed of WT and vCT zebrafish peaked during the first 10 s of 2 min light stimulation, whereas that of 4vMO zebrafish did not have a peak value (Fig. 6A). Compared to the average swimming speed during 2 min dark treatment, the average velocity in the first 10 s of the light stimulation was significantly higher in the WT (*P* < 0.001) and vCT zebrafish (*P* < 0.01) (n ≥ 40, Fig. 6B). However, there was no significant difference in the average swimming speed of 4vMO zebrafish between two periods (*P* > 0.05, n ≥ 40, Fig. 6B). Following these findings, *lhx4* knockdown in the retina via 4vMO was concluded to weaken the light stimulation response.

## Discussion

Based on previous reports, LHX4 is expressed in BCs and PRs of both mouse and chicken retinas. In mice, LHX4 is transiently expressed in the rod BCs and RPs of the developing retina and persistently expressed in the CPs, ON-cone BCs, and four subtypes of OFF-cone BCs (type 2, 3a, 3b, and 4) of the adult retina^34–36^. Buenaventura et al. found that the conservation expression of LHX4 in early CPs of mouse and chicken retinas is a new marker of early CPs^37^. Here, *lhx4* expression was detected in the zebrafish retina for the first time using ISH, and it was continuously expressed in the ONL and INL. A lack of relevant antibodies poses challenges in locating the definitive cell types that express *lhx4* in the zebrafish retina.

Of note, RGCs were first withdrawn from the cell cycle in the zebrafish retina and then differentiated in the nasal-dorsal regions at 28 hpf, followed by PRs, ACs, HCs, and BCs. Then, MGCs began to differentiate at 60 hpf. At this time, the retina was stratified to form the above-mentioned five-layered structure. In zebrafish, vision worked at 72 hpf, and the functional visual behavior experiments could be carried out at 96 hpf^39^. Our results showed *lhx4* expression was initiated in the retina at 48 hpf, indicating that *lhx4* might play an essential role in zebrafish retinal development.

Gene knockdown via MO has been widely used in zebrafish for many years^40–42^. An Lhx4 translation-blocking MO was previously employed to explore the role of Lhx4 in the fates of spinal cord interneurons^33^. Herein, *lhx4* expression was knocked-down via a splice-blocking MO. Subsequent results showed that under the optimal MO injection dose (2 ng), the frameshift mutation (deletion of exon 2) of *lhx4* mRNA resulted in a significant decrease of body length and eye size, abnormal retinal structure, and neural differentiation without affecting apoptosis in the retina, and impairing visual function and swimming ability of zebrafish. Lhx4 is critical to pituitary development^43, 44^. Besides, the mutation of human *LHX4* is usually associated with CPHD (characterized by a deficiency of the growth hormone and growth retardation)^32, 45, 46^. As a result, we speculated that *lhx4* knockdown via MO in zebrafish is phenotypically similar to human CPHD. These results indicate that this *lhx4* knockdown model via MO is unsuitable for investigating the role of *lhx4* in zebrafish retinal development. Moreover, we injected the *lhx4 *vivo-MO into the left eyes at 26–27 hpf to knockdown the expression of *lhx4* in the eyes of zebrafish.

The injection dose of vivo-MO in vivo is relatively high^47, 48^. Herein, we induced frameshift mutation of *lhx4* mRNA in zebrafish eyes at 5 ng dosage. In a previous report, when Chen et al. injected Protocadherin-17 vivo-MO into the right eyes of zebrafish at 25–26 hpf, the phenotype of the left eyes was similar to that of the right eyes at 49 hpf and 72 hpf. The results indicate that vivo-MO injected into one eye could be transmitted to the opposite eye through the circulatory system^49^. It was revealed that *lhx4 *vivo-MO injected into the left eyes potentially triggered the knockdown of *lhx4* mRNA expression in both the left and right eyes. Consequently, we injected *lhx4 *vivo-MO into the left eyes of zebrafish to assess its role in retinal development. Subsequent experiments proved that the abnormal phenotype of zebrafish was caused by *lhx4* knockdown through MO due to the vital function of *lhx4* in pituitary development. The results obtained with 4MO and 4vMO were different, which may be due to the different injection times and sites; 4vMO was injected into one eye of the embryo at 26–27 hpf, whereas 4MO was injected into the yolk sac at the 1- to 4-cell stage. Of note, 4MO could knock down the expression of *lhx4* in the whole embryo, but 4vMO knocked down the expression of *lhx4* in the eyes. However, due to the limitation of this MO-based approach and the lack of suitable antibodies in zebrafish, we could not demonstrate the actual 4vMO distribution in the eye tissue layers and the effect of *lhx4* knockdown.

In recent years, an increasing number of studies on light stimulus responses have been conducted to investigate the visual function in zebrafish^50–53^. Zebrafish typically display phototaxis behaviors, and they rapidly swim towards the light from the dark. Thus, their swimming speed increases dramatically within the first 10 s of the light stimulus. Previous assessments found that the visual function in zebrafish forms at 4 dpf, whereas the swimming ability develops at 5 dpf^54, 55^. Furthermore, Deiodinase 3 knockdown by MO weakens the responses to a light stimulus in zebrafish at 4 dpf and 5 dpf^52^. Compared to WT zebrafish, IL7R mutant zebrafish displays slower light stimulation responses at 6 dpf^53^. In the present study, testing the responses to a light stimulus at 5 dpf revealed that zebrafish with *lhx4* knockdown via 4vMO in the eyes had weakened light stimulation responses, damaging their visual function. Similar to Cai et al.^53^, the present findings showed that the swimming speed of all larvae was similar under light, whereas the average swimming speed of 4MO and 4vMO larvae is higher than that of WT and vCT larvae in the dark. Thus, we presumed that the larvae could not receive light signals or get anxious in the dark due to the absence of RPs.

Also, we found that *lhx4* knockdown in zebrafish eyes reduces the number of Parvalbumin^+^ ACs and Rhodopsin^+^ RPs significantly, increases the expression of *vsx2*, and damages the visual function of the larvae. Of note, no effect occurred on the differentiation of OFF-BCs (marked by *vsx1*), rod BCs (marked by PKCα and *bhlhe23*), and apoptosis (marked by activated-Caspase3) in the retina. In mice, Dong et al. found that Lhx4 CKO (conditional knockout) results in the loss of rod BCs, rod-connecting BCs, and AII ACs, and a visual defect resembling the human CSNB (congenital stationary night blindness)^34^. Together, these findings demonstrate that Lhx4 plays an important role in vertebrate retinal development.

Vsx2 is expressed in the RPCs of the entire retinal epithelium at the early stage of zebrafish retinal development^9^. These pluripotent RPCs potentially differentiate into various retinal cell types. Withdrawing RPCs from the cell cycle downregulated Vsx2 expression. Further, the expression of various cell-specific transcription factors was activated, thereby differentiating into different types of retinal cells^2, 10^. Moreover, Vsx2 overexpression elevates BC levels at the expense of RPs in the mouse retina^56^. Therefore, we speculated that lhx4 knockdown via vivo-MO in the eyes accelerates the differentiation of ON-cone BCs. It is assumed that these reduced retinal cells may be transformed into excessive ON-cone BCs through overexpression of *vsx2.*

However, Seredick et al. observed a reduced number of Vsx2^+^ cells in the absence of Lhx4 in the spinal cord of older embryos^33^. In the mouse retina, Dong et al. found the expression of VSX2 is not changed in the early retina but decreased through apoptosis in *Lhx4* null mice at the later stages^34^. Zebrafish *vsx2* mRNA contains 5 exons; the primer pair used above is located in the E1 and E4. To validate our findings, we designed four primer pairs to analyze the expression change in different regions of *vsx2* mRNA, including E2-E3, E3-E4, E4-E5, and E2-E4. Compared to WT and vCT groups, the expression of these regions was significantly higher in 4vMO retinas at 60 hpf (Fig. S10). We believe that the reasons for the paradox are as follows: First, Lhx4 knockdown via vivo-MO did not cause apoptosis in the zebrafish retina. Besides, the expression of Vsx2 protein and mRNA may not be consistent.

In conclusion, the present study revealed that *lhx4* regulates neural differentiation in the retina and visual function during zebrafish embryonic development. However, the effects of *lhx4* knockdown in the eyes on MGCs, cone BCs, and cell proliferation were not detected. Moreover, the mechanism by which Vsx2 overexpression is induced via Lhx4 knockdown and its regulatory roles in the differentiation of the various retinal cell types are yet to be elucidated. Further studies will be carried out in the future, such as the construction of a zebrafish model of Lhx4 conditional knockout by CRISPR-Cas9, to validate the function of Lhx4 in vertebrate retinal development.

## Materials and methods

### Zebrafish maintenance

Zebrafish (WT/AB) were maintained as reported by Sheng et al.^57^. Embryos were collected, reared in E3 medium, and staged for hours post-fertilization (hpf) or days post fertilization (dpf). All animal experiments were performed under the Animal Use and Care Committee of Hangzhou Normal University, Hangzhou, China and approved by the Animal Use and Care Committee of Hangzhou Normal University, Hangzhou, China.

### ISH

The embryos were fixed in 4% paraformaldehyde (PFA) at 4 ˚C, dehydrated in 30% sucrose, and cryosectioned at 16 μm thickness. The procedures for ISH were as described in our previous study^58^.

### MO injection

Four MOs were obtained from Gene Tools (USA), including splice-blocking *lhx4* MO (named as 4MO), *lhx4 *vivo-MO (named as 4vMO) (TGT AGA AGA GCT GTA CTG ACT TGA A), standard control MO (named as CTMO), and vivo-MO (named as vCTMO) (CCT CTT ACC TCA GTT ACA ATT TAT A). 4MO or CTMO was injected into the embryos of 4MO or CT group at 1-to 4-cell stage. 4vMO or vCTMO was injected into one eye of 4vMO or vCT embryos at 26–27 hpf. The WT embryos were raised without any treatment.

We confirmed the efficiency of 4MO or 4vMO 24 h post-injection (hpi). The total RNA was extracted from whole embryos at 36hpf or the eyes at 60 hpf with TRIzol reagent (Sangon, China). Complementary DNA (cDNA) was synthesized by Prime Script Reverse Transcriptase System Kit (Takara, Japan). The primers F: TCTGCCGGACATCCTCG and R: CTCTCATGTCCAGGCCCGTTT, which span from exon 1 to exon 4 of the *lhx4* transcript, were used to amplify the sequence region by reverse transcription-polymerase chain reaction (RT-PCR).

### Morphometric analysis

Images of zebrafish embryos or larvae were captured under a dissecting microscope (Digital sight; Nikon, Japan). The distance from the epiphysis to the tail tip was measured as body length, along the anterior–posterior axis. The whole eye, including the lens, was outlined as the eye area^52, 53^.

### Immunofluorescence staining

The embryos were pre-treated using the above ISH experiment. Other procedures were performed according to the previous report^59^. The primary antibodies used in this study are listed in Table S1.

### Quantitative real-time PCR (qRT-PCR)

cDNA from the whole embryo or eye was obtained at 60 hpf, as described by He et al*.*^60^ Primers are listed in Table S2.

### Responses to the light stimulus

The larvae of the different groups were placed in a 96-well plate at 5 dpf to test the responses to the light stimulus with a Danio Vision system (Noldus Information Technology, Netherlands)^52, 53^. Larvae were placed in the dark for a 30-min adaption and then subjected to a 30-min light stimulus. The average speed of larvae was calculated for 4 min, including the last 2 min in the dark and the first 2 min in light.

### Statistical analysis

The number of positive cells in each image was counted manually via immunofluorescence staining. Notably, we counted 2–4 sections per embryo. Statistical analysis was performed in GraphPad Prism software version 8.0.1 (https://www.graphpad.com). A one-way analysis of variance (ANOVA) was performed for multiple comparisons. The error bar in graphs represents the standard error of the mean (SEM), whereas “n” denotes the number of zebrafish examined. *P-*value < 0.05 is defined as statistically significant. *P < 0.05, **P < 0.01, ***P < 0.001, ****P < 0.0001.

## Supplementary Information

Supplementary Information

## Data Availability

All data generated or analyzed during this study are included in this published article (and its Supplementary Information files).
