# Author Correction: *LIM Homeobox 4* (*lhx4*) regulates retinal neural differentiation and visual function in zebrafish

**DOI:** 10.1038/s41598-025-90120-1

**Published:** 2025-04-15

**Authors:** Rui Guo, Kangkang Ge, Yuying Wang, Minxia Lu, Fei Li, Lili Tian, Lin Gan, Donglai Sheng

**Affiliations:** 1 College of Life Sciences, Zhejiang University, Hangzhou, 310013 Zhejiang China; 2 Key Laboratory of Organ Development and Regeneration of Zhejiang Province, College of Life and Environmental Sciences, Hangzhou Normal University, Hangzhou, 311100 Zhejiang China; 3 Hangzhou Jingbai Biotechnology Co, LTD, Hangzhou, 310004 Zhejiang China; 4 Traditional Chinese Medicine Pharmacy, Zhejiang Hospital, Hangzhou, 310007 Zhejiang China

Correction to: *Scientific Reports* 10.1038/s41598-021-81211-w, published online 21 January 2021

This article contains an error in Figure 5, where the image of the WT group is mistakenly cited as the image of the vCT group (marked as WTv due to inconsistent initial experimental labeling) in the organization of panel (d).

The correct Figure 5 and accompanying legend appear below.

**Fig. 5:**
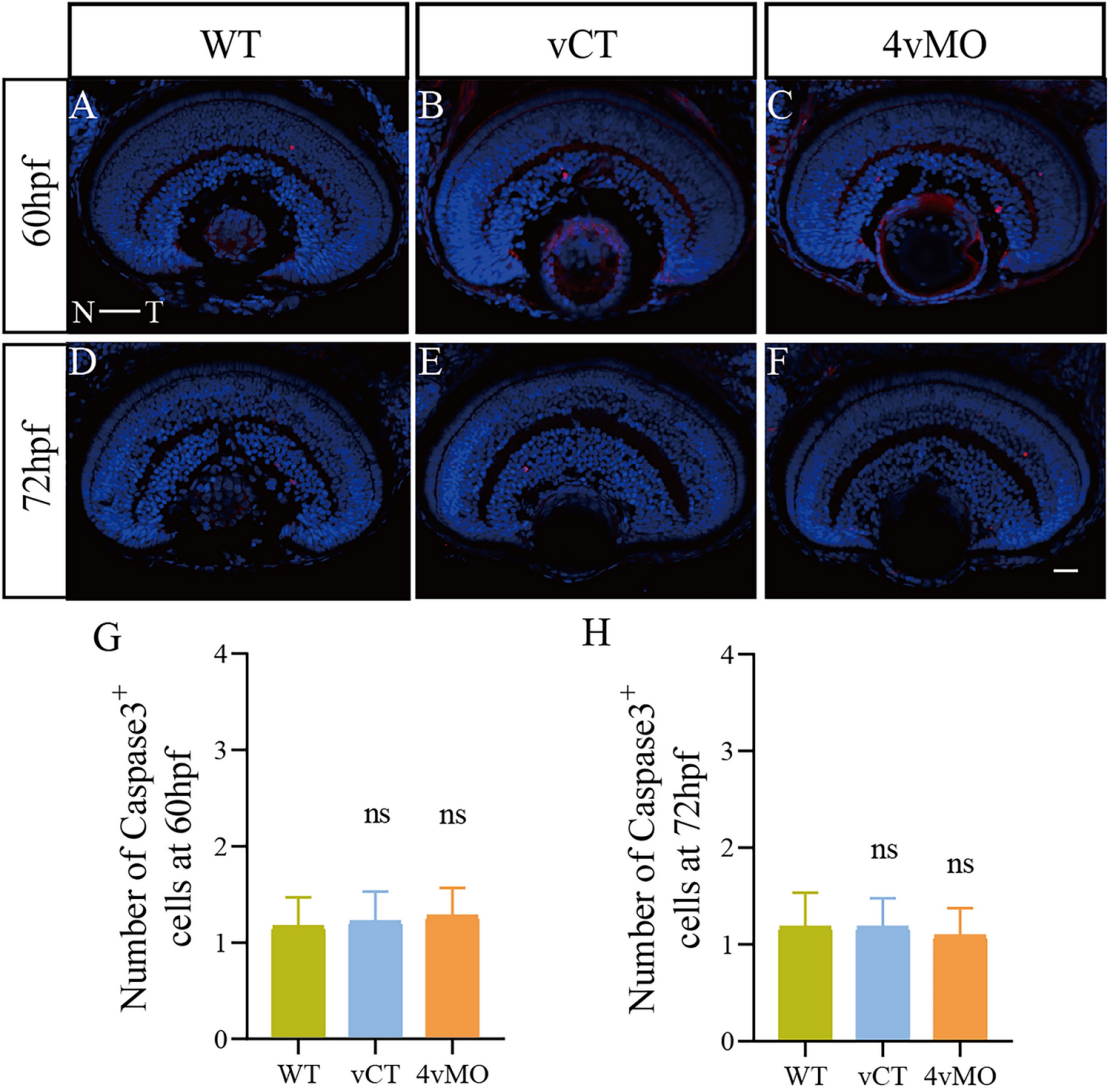
Effects of *lhx4* knockdown via vivo-MO in the eyes on the apoptosis in the retina. All figures are horizontal sections along the temporal-nasal axis (T-N). (**A**–**F**) Immunofluorescence staining with Caspase3 at 60 hpf and 72 hpf. Blue, DAPI staining of the nuclei. Scale bar = 20 μm. (**G**,**H**) Statistical analysis of the number of Caspase3^+^ cells in WT, vCT, and 4vMO retinas at 60 hpf and 72 hpf. ns, *P* > 0.05; vCT vs. WT; 4vMO vs. vCT. Results are presented as the mean ± SEM (n ≥ 10).

