# Supplementary material for: LIM Homeobox 4 (lhx4) regulates retinal neural differentiation and visual function in zebrafish
Source: Sci Rep. 2021 Jan 21;11:1977. doi: 10.1038/s41598-021-81211-w (PMC7820405; doi:10.1038/s41598-021-81211-w)
Supplement: Supplementary file 1 — Supplementary Information [file 41598_2021_81211_MOESM1_ESM.docx]

***LIM Homeobox 4* (*lhx4*) Regulates Retinal Neural Differentiation and Visual Function in Zebrafish**

**Rui Guo^a,b^, Kangkang Ge^c^, Yuying Wang^b^, Minxia Lu^b^, Fei Li^b^, Lili Tian^d^, Lin Gan^a,b,*^, Donglai Sheng^b,*^**

^a^ *College of Life Sciences, Zhejiang University, Hangzhou, 310013, Zhejiang, China*

^b^ *Key Laboratory of Organ Development and Regeneration of Zhejiang Province, College of Life and Environmental Sciences,* *Hangzhou Normal University, Hangzhou, 311100, Zhejiang, China*

*^c^* *Hangzhou jingbai biotechnology Co, LTD. Hangzhou, 310004, Zhejiang, China*

*^d^* *Traditional Chinese Medicine Pharmacy, Zhejiang Hospital, Hangzhou,* *310007, Zhejiang, China.*

^*^ **Corresponding author：**

Donglai Sheng, Key Laboratory of Organ Development and Regeneration of Zhejiang Province, College of Life and Environmental Sciences, Hangzhou Normal University, Hangzhou, 311100, Zhejiang, China. Email: sheng_dl@hotmail.com

Lin Gan, College of Life Sciences, Zhejiang University, Hangzhou, 310013, Zhejiang, China. Email: lin_gan@idrbio.org

## Supplemental Materials

**Table S1** Primary antibodies for immunofluorescence staining

| Antibody | Dilution | Source | Recognization |
| --- | --- | --- | --- |
| Rb anti- GABA | 1:500 | Sigma | GABAergic ACs |
| Ms anti- Parvalbumin | 1:50 | Sigma | One ACs type |
| Rb anti-TH | 1:50 | Chemicon | dopaminergic ACs |
| Rb anti- Calretinin | 1:200 | Millipore | One ACs type |
| Ms anti- Zn-8 | 1:10 | DSHB | RGCs |
| Rb anti- PKCα | 1:1000 | Sigma | On-BCs |
| Ms anti- Zpr-1 | 1:500 | Abcam | Red and green cones |
| Ms anti- Rhodopsin | 1:500 | Abcam | rods |
| Rb anti- Activated Caspase-3 | 1:200 | R&D Systems | apoptosis cells |

**Table S2** Primers for qRT-PCR

| Gene name | Primer sequence |
| --- | --- |
| *vsx1* F | TCAGGGAACTCTCAAAAGAGGAAAAA |
| *vsx1* R | ACCTGTATCCTGTCCTCTGGTAGCTCT |
| *vsx2* F | 5’-TCTTTCTACAGTCAGCCCGC-3’ |
| *vsx2* R | 5’-GTGCATCCCTAGAAGCCAGG-3’ |
| *prox1* F | 5’-ACCATGACAGCACATCCCTC-3’ |
| *prox1* R | 5’-ACGTTGGACTTCTCACCGTC-3’ |
| gs F | 5’-CACGTCTGCCAGTTCTCAGT-3’ |
| *gs* R | 5’-GCCTTCAGCTTGATACGTGC-3’ |
| *bhlhe23* F | AAGGACAGACTATAACTTCGCCGATTC |
| *bhlhe23* F | TATCGTTGCAGTGTTTGAACAGGT |
| *β-actin* F | 5’- CATGCCATCCTGCGTCT-3’ |
| *β-actin* R | 5’- AAACGCTCATTGCCGAT-3’ |
| *vsx2* E2E3-F | 5’-AGTCTTCACTGAGCCAGAGC-3’ |
| *vsx2* E2E3-R | 5’-TTCTCTGGCGTACACATCCG-3’ |
| *vsx2* E3E4-F | 5’-CGGATGTGTACGCCAGAGAA-3’ |
| *vsx2* E3E4-R | 5’-CGTATTCCGCCATCACGCTA-3’ |
| *vsx2* E4E5-F | 5’-GCGTCACTCTATCCCTCTGC-3’ |
| *vsx2* E4E5-R | 5’-TTGGGATTGGTCGGTGTCTG-3’ |
| *vsx2* E2E4-F | 5’-CACTGAGCCAGAGCAAGAAGA-3’ |
| *vsx2* E2E4-R | 5’-CTTGGCCCTCCGGTTTTGA-3’ |


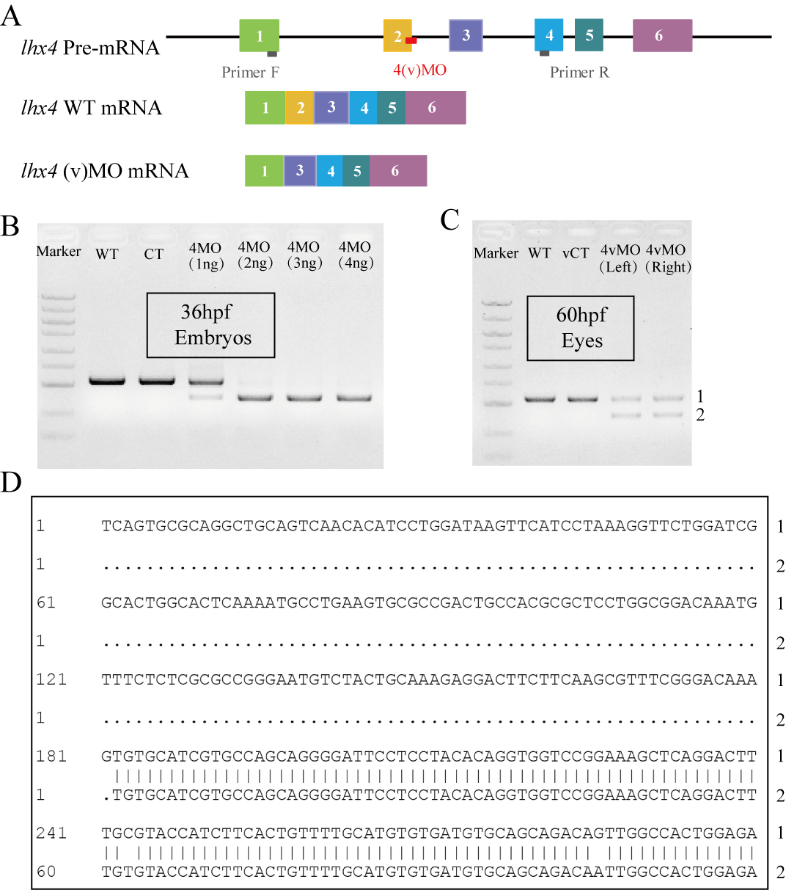


**Figure S1.** Identification of *lhx4* (*vivo*-) MO efficacy. (A) Schematic diagram of *lhx4* (*vivo*-)MO blocking *lhx4* mRNA splicing. (B) The effect of splice-blocking *lhx4* MO on *lhx4* transcript at 36 hpf by RT-PCR. WT, CT, and 4MO lanes used cDNA from WT, CT, and 4MO embryos as templates. (C) The effect of splice-blocking *lhx4* *vivo*-MO on *lhx4* transcript in the eyes at 60 hpf by RT-PCR. WTv, vCT, and 4vMO lanes used cDNA from WT, vCT, and 4vMO groups as templates. (D) Sequence alignment of band 1 (551 bp) and band 2 (379 bp).


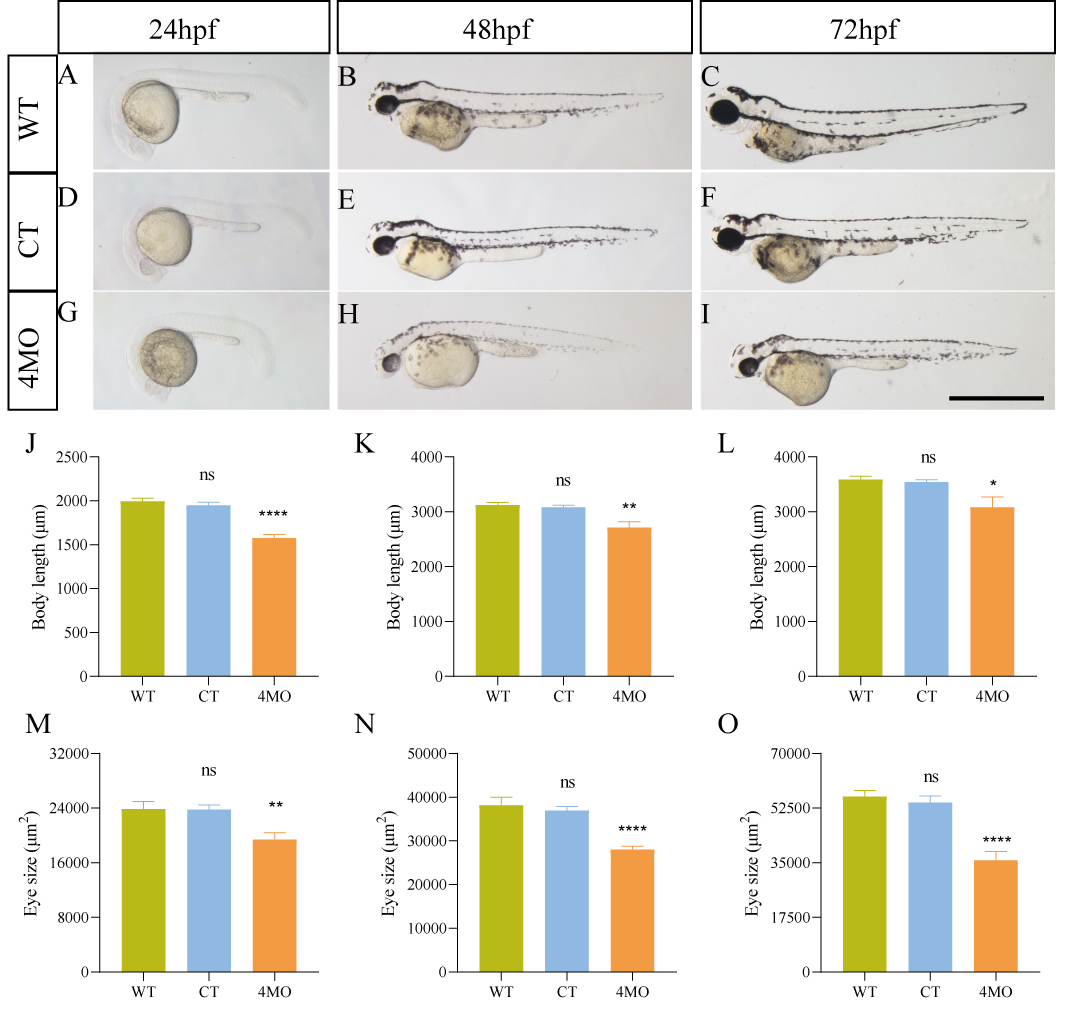


**Figure S2.** Effects of *lhx4* knockdown via MO on the zebrafish morphology and eye development. (A-I), Lateral view of WT, CT, and 4MO embryos at 24 hpf, 48 hpf, and 72 hpf. Scale bar = 1000 μm. (J-O) Graphic analysis of body length and eye size of embryos. ns, *P* > 0.05; *, *P* < 0.05; **, *P* < 0.01; ****, *P* < 0.0001; CT vs. WT; 4MO vs. CT. Results are presented as the mean ± SEM (n ≥ 15).


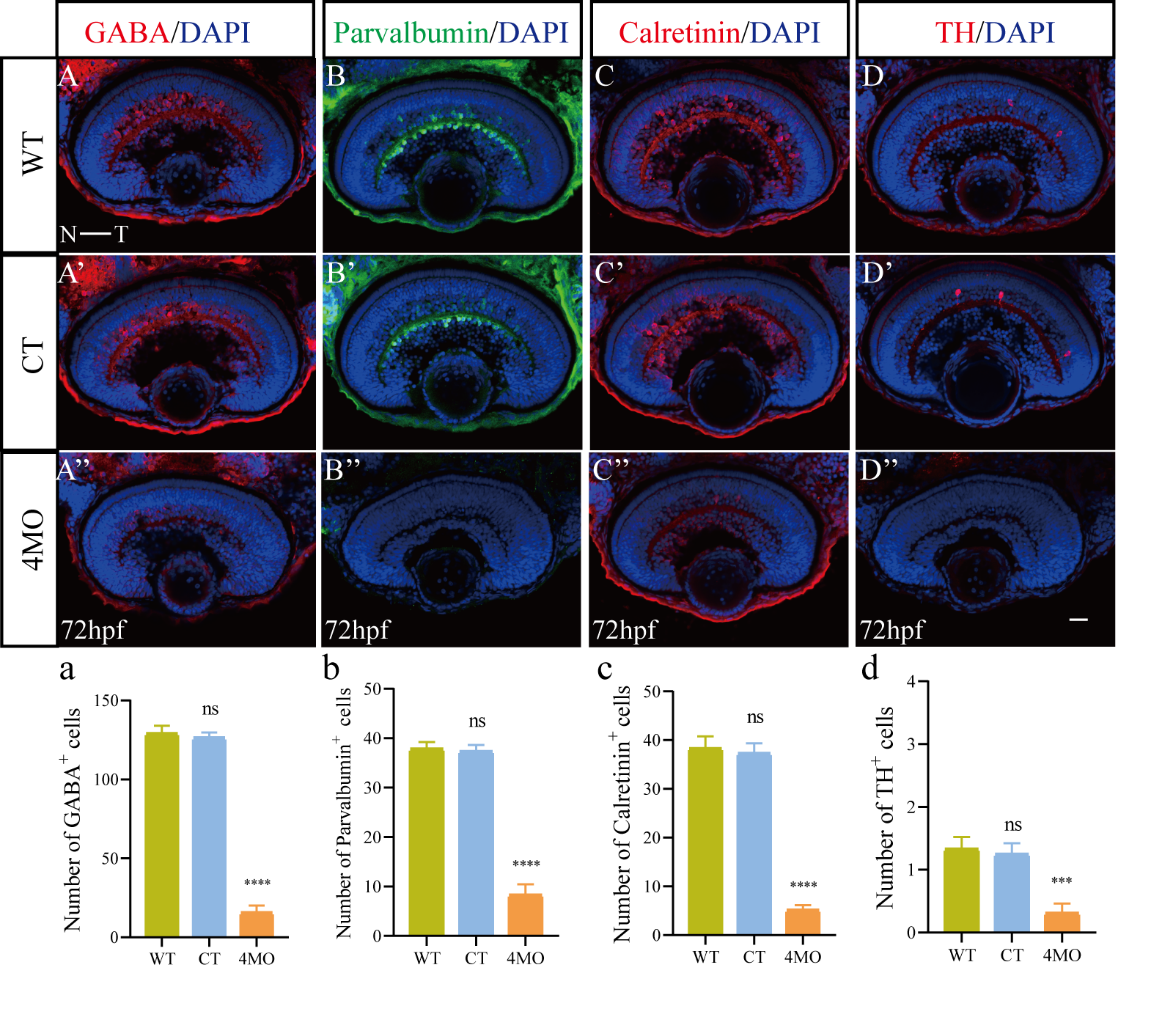


**Figure S3.** Effects of *lhx4* knockdown via MO on the ACs differentiation. All figures are horizontal sections along the temporal-nasal axis (T-N). (A-D) Immunofluorescence staining with different ACs markers, GABA, Parvalbumin, Calretinin, and TH, in WT, CT, and 4MO retinas at 72 hpf. Blue, DAPI staining of the nuclei. Scale bar = 20 μm. (a-d) Statistical analysis of GABA^+^, Parvalbumin^+^, Calretinin^+^, and TH^+^ cells in WT, CT, and 4MO retinas at 72 hpf. ns, *P* > 0.05; ***, *P* < 0.001; ****, *P* < 0.0001; CT vs. WT; 4MO vs. CT. Results are presented as the mean ± SEM (n ≥ 10).


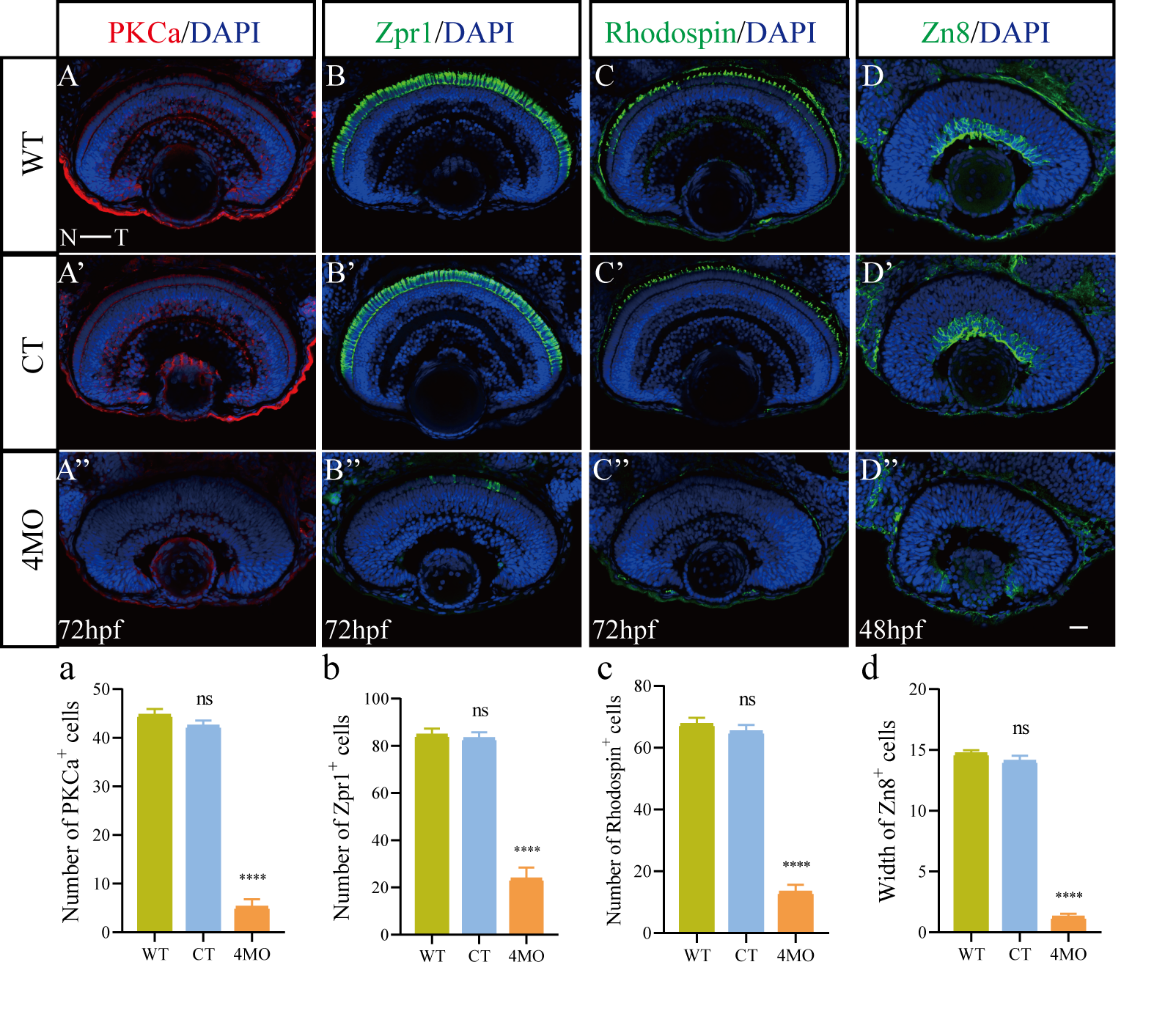


**Figure S4.** Effects of *lhx4* knockdown via MO on the other retinal neuronal differentiation. All figures are horizontal sections along the temporal-nasal axis (T-N). (A-D) Immunofluorescence staining with other retinal neural markers, PKCα (at 72 hpf), Zpr1 (at 72 hpf), Rhodopsin (at 72 hpf), and Zn8 (at 48 hpf) in WT, CT, and 4MO retinas. Blue, DAPI staining of the nuclei. Scale bar = 20 μm. (a-d) Statistical analysis of PKCα^+^, Zpr1^+^, Rhodospin^+^, and Zn8^+^ cells in WT, CT, and 4MO retinas. ns, *P* > 0.05; ****, *P* < 0.0001; CT vs. WT; 4MO vs. CT. Results are presented as the mean ± SEM (n ≥ 10).


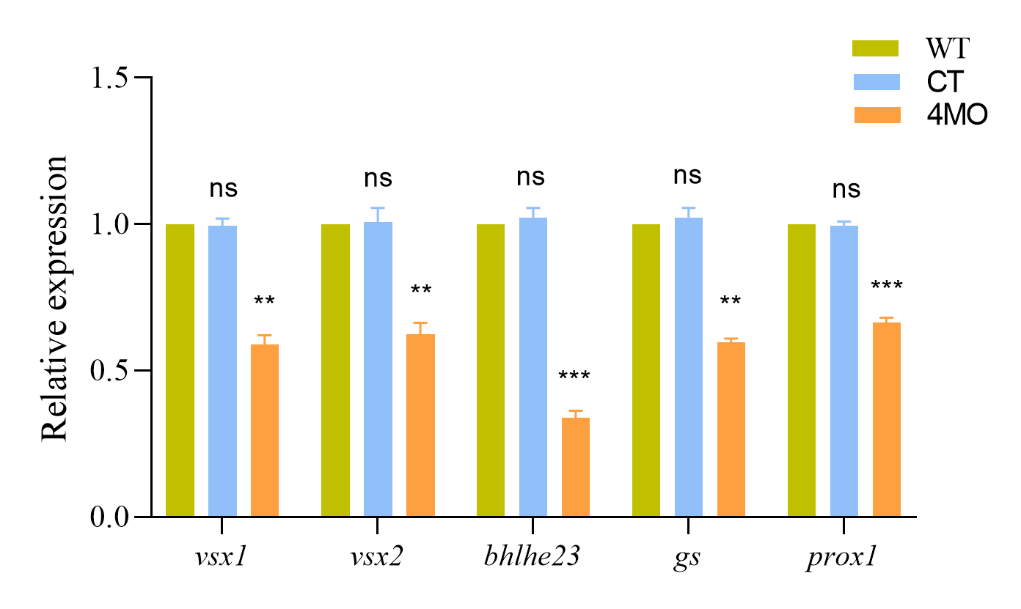


**Figure S5.** Effects of *lhx4* knockdown via MO on the expression of other retinal genes at 60hpf. ns, *P* > 0.05; **, *P* < 0.01; ***, *P* < 0.001; CT vs. WT; 4MO vs. CT. Results are presented mean ± SEM (n = 3 biological replicates).


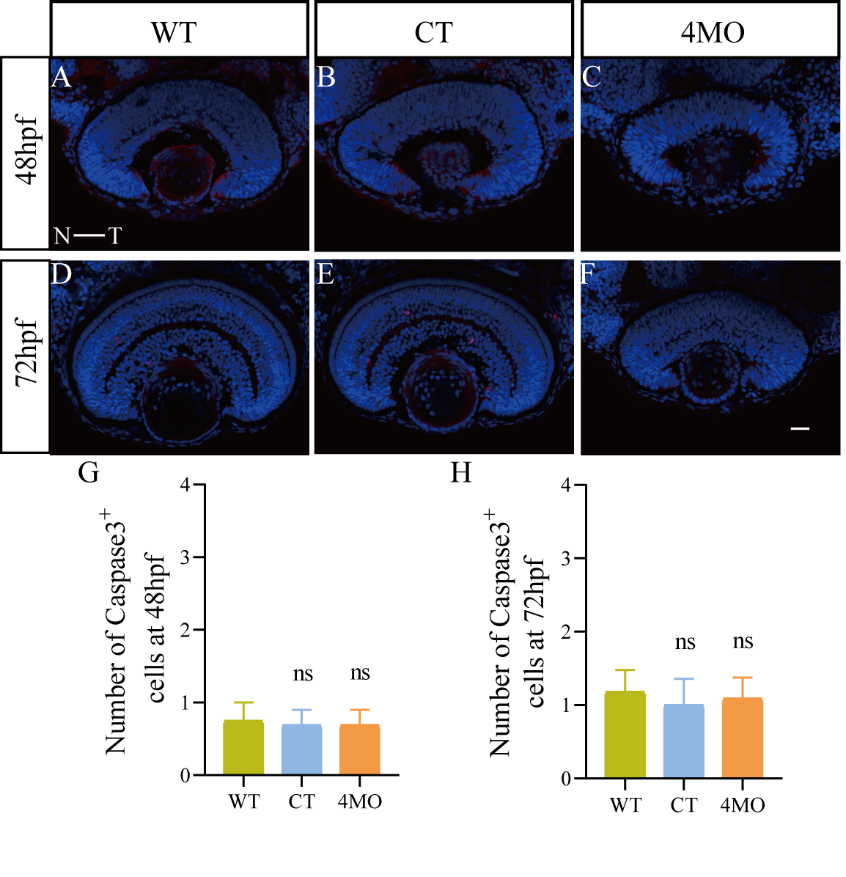


**Figure S6.** Effects of *lhx4* knockdown via MO on the apoptosis in the retina. All figures are horizontal sections along the temporal-nasal axis (T-N). (A-F) Immunofluorescence staining with Caspase3 at 48hpf and 72hpf. Blue, DAPI staining of the nuclei. Scale bar = 20 μm. (G-H) Statistical analysis of the number of caspase3^+^ cells in WT, CT, and 4MO retinas at 48hpf and 72hpf. ns, *P* > 0.05; CT vs. WT; 4MO vs. CT. Results are presented as the mean ± SEM (n ≥ 10).


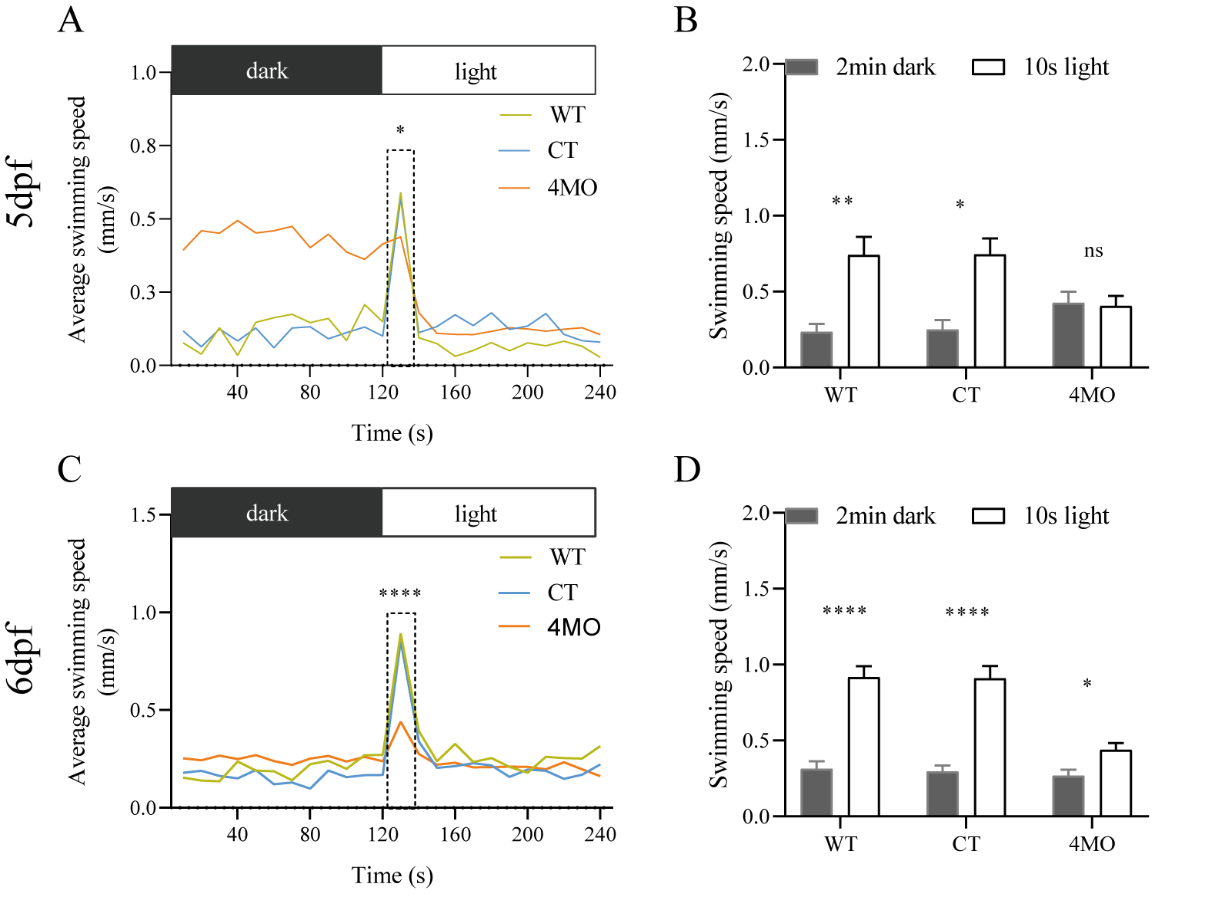


**Figure S7.** Effects of *lhx4* knockdown via MO on the visual function and swimming ability of zebrafish. (A) The average swimming speed of the larvae during the last 2min dark and 2min light period. (B) The swimming speed during the last 2min dark and the first 10s of the 2min light period. ns, *P* > 0.05; *, *P* < 0.05; **, *P* < 0.01; CT vs. WT; 4MO vs. CT. Results are presented as the mean ± SEM (n ≥ 40).


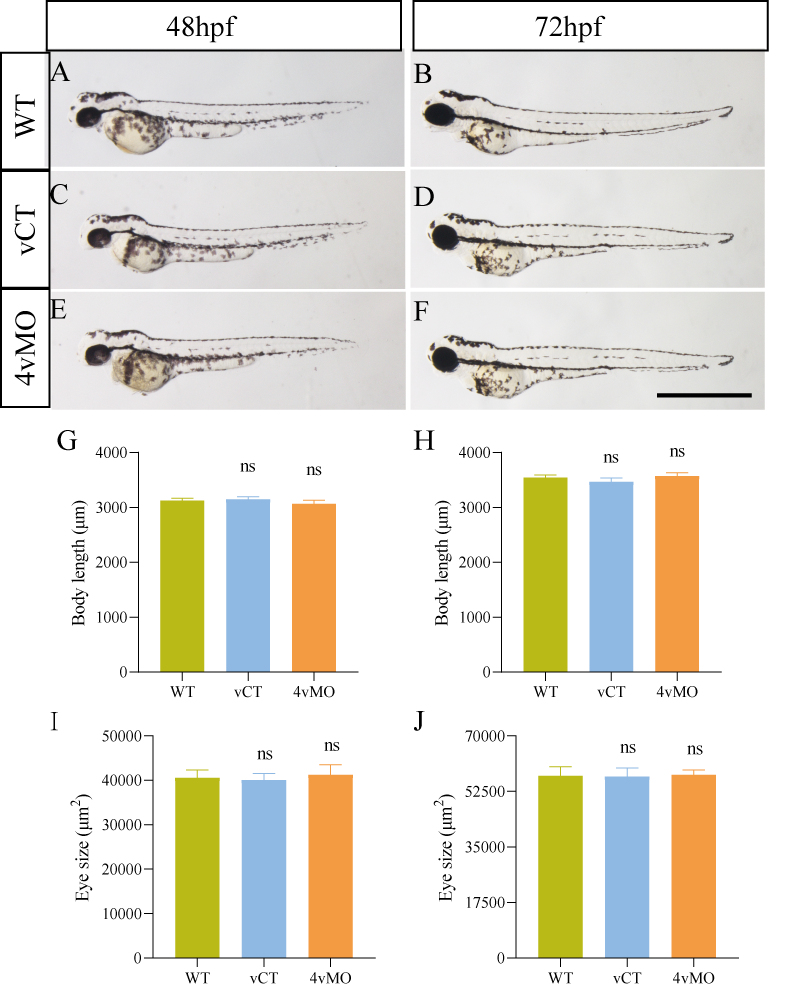


**Figure S8.** Effects of *lhx4* knockdown via *vivo-*MO in the eyes on the zebrafish morphology and eye development. (A-I) Lateral view of WT, vCT, and 4vMO embryos at 48hpf and 72hpf. Scale bar = 1000 μm. (G-J) Graphic analysis of body length and eye size of embryos. ns, *P* > 0.05; vCT vs. WT; 4vMO vs. vCT. Results are presented as the mean ± SEM (n ≥ 15).


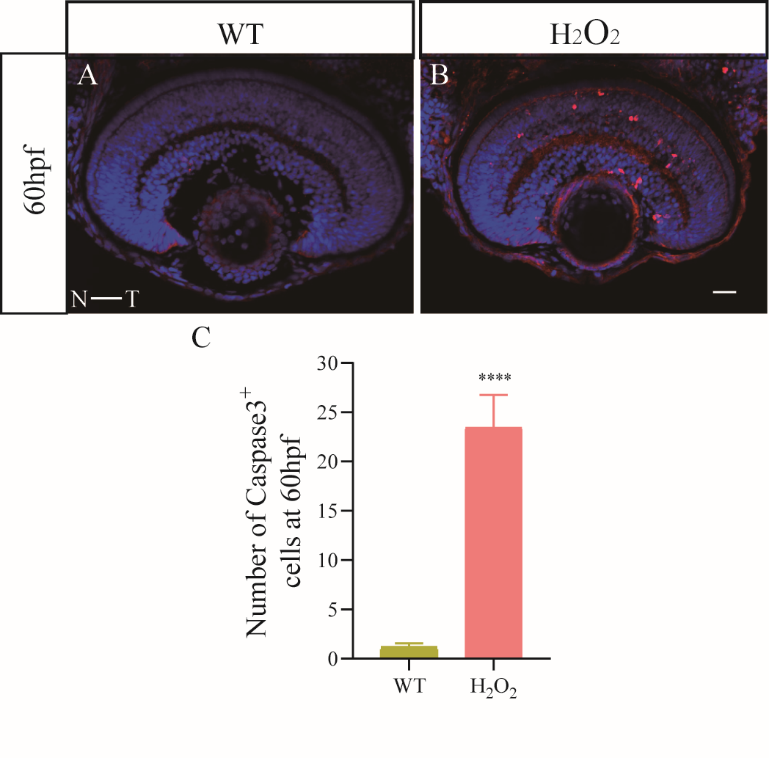


**Figure S9.** The apoptosis in the retina with zebrafish treated with 2 mM H_2_O_2_ for 6 h. All figures are horizontal sections along the temporal-nasal axis (T-N). (A-B) Immunofluorescence staining with Caspase3 at 60 hpf. Blue, DAPI staining of the nuclei. Scale bar = 20 μm. (C) Statistical analysis of the number of Caspase3^+^ cells in WT and retinas at 60 hpf. ****, *P* < 0.0001; H_2_O_2_ vs. WT. Results are presented as the mean ± SEM (n ≥ 10).


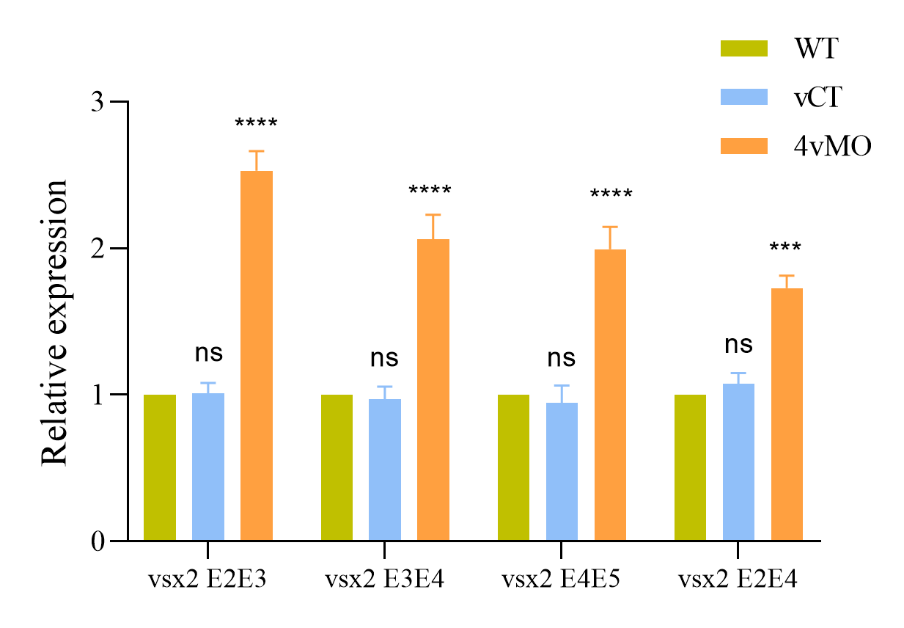


**Figure S10.** Effects of *lhx4* knockdown via *vivo-*MO in the eyes on the expression of different regions in *vsx2* mRNA at 60 hpf. ns, *P* > 0.05; ***, *P* < 0.001; ****, *P* < 0.0001; vCT vs. WT; 4vMO vs. vCT. Results are presented mean ± SEM (n = 3 biological replicates).
